# The Effects of Astaxanthin on Metabolic Syndrome: A Comprehensive Review

**DOI:** 10.3390/md23010009

**Published:** 2024-12-27

**Authors:** Chunhao Gao, Nengyun Gong, Fangtian Chen, Shiran Hu, Qingxin Zhou, Xiang Gao

**Affiliations:** 1 College of Life Sciences, Qingdao University, Qingdao 266071, China; gaoch0306@163.com (C.G.); gny907@126.com (N.G.); hushiran0131@163.com (S.H.); 2 Department of Marine Technology, Rizhao Polytechnic, Shandong Engineering and Technology Research Center for Marine Crustacean Resources Comprehensive Utilization, Shandong Engineering Research Center for Efficient Utilization Technology of Marine Food Resources, Rizhao Key Laboratory of Efficient Utilization of Marine Food Resources, Rizhao 276826, China; fangxitianjian@126.com

**Keywords:** astaxanthin, metabolic syndrome, obesity, insulin resistance, dyslipidemia

## Abstract

Metabolic syndrome (MS) represents a complex cluster of metabolic disorders primarily characterized by obesity, insulin resistance, hyperglycemia, dyslipidemia, hypertension, and hyperuricemia. Diet and functional ingredients play a pivotal role in seeking non-pharmacological strategies to prevent and ameliorate MS. Astaxanthin (AST), a carotenoid found in various marine organisms, exhibits exceptional antioxidant properties and holds great promise as a natural compound that improves MS. This article introduces the basic properties of AST, including its absorptance and metabolic pathways, along with various isomers. Most importantly, we comprehensively review the effects and mechanisms of AST on improving the primary components of MS. These mechanisms primarily involve regulating signal transduction, transport, or metabolic pathways within the body, as well as influencing intestinal microbiota and metabolites, thereby exerting positive effects on metabolism and inhibiting the occurrence of MS. This review emphasizes the potential efficacy of AST in managing MS. However, more studies are needed to confirm the clinical effect of AST on MS and reveal potential molecular mechanisms.

## 1. Introduction

Metabolic syndrome (MS), also known as syndrome X, is a complex and multifaceted health problem involving the intersection of multiple cardiovascular and metabolic diseases [1,2]. A Swedish physician first introduced the concept of MS in the 1920s. However, it was not until 1998 that the World Health Organization (WHO) established a preliminary unified definition for MS [3]. In the subsequent years, the definition of MS underwent refinement. The current widely accepted definition is from the International Diabetes Federation (IDF) in 2005 [4,5], which defines MS as a clinical syndrome characterized by central obesity as a core element and highly associated with type 2 diabetes and cardiovascular diseases. The diagnostic criteria provided by the IDF are as follows: (a) waist circumference: male ≥90 cm, female ≥80 cm; (b) triglycerides (TG): ≥1.7 mmol/L (or 150 mg/dL); (c) high-density lipoprotein cholesterol (HDL-C): male <1.03 mmol/L (or 40 mg/dL), female <1.29 mmol/L (or 50 mg/dL); (d) hypertension: Systolic blood pressure ≥ 130 mmHg or diastolic blood pressure ≥ 85 mmHg; and (e) hyperglycemia: fasting blood glucose ≥ 5.6 mmol/L (or 100 mg/dL). A diagnosis of MS can be made if two or more of the above criteria are met [6]. The global prevalence of MS is around 30%, with developed countries such as the United States having a higher prevalence. Epidemiological data show that the prevalence of MS among adults in the United States increased from 36.2% in 1999–2000 to 47.3% in 2017–2018 [7,8]. MS increases the risks of sudden cardiac death by 70%, cardiovascular disease by two times, and type 2 diabetes mellitus (T2DM) by five times, thereby increasing healthcare costs [9,10,11]. Medications alone or in combination with regular exercise and dietary control are commonly utilized approaches to manage MS. However, medications usually have adverse effects, and patients are often unable to achieve the recommended minimum level of exercise and have difficulty following a dietary regimen [12,13]. Thereby, the utilization of natural biologically active compounds, nutraceuticals, and supplements has emerged as an effective and economical way to prevent and treat MS [14,15].

**Figure 1:**
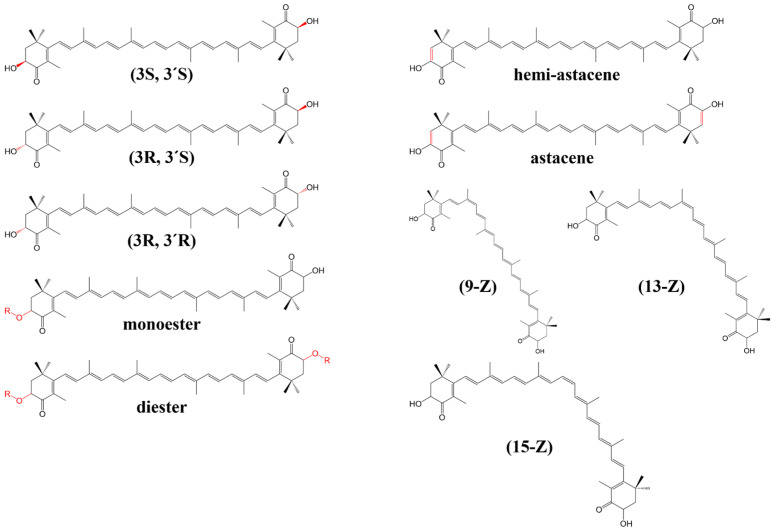
Structures of AST isomers.

Astaxanthin (AST), a xanthophyll carotenoid, has garnered tremendous attention in recent years due to its excellent physical, biochemical, and physiological properties. AST has the strongest anti-oxidative properties among carotenoids, which are 14-, 65-, and 54-fold higher than the antioxidant capacity of vitamin E, vitamin C, and β-carotene, respectively [3,16]. Compared to other carotenoids, the intestinal absorptive efficacy of astaxanthin is at a moderate level, which is weaker compared to β-carotene, α-carotene, β-cryptoxanthin, canthaxanthin, lutein, and zeaxanthin but stronger than fucoxanthin and neoxanthin [17]. As presented in Figure 1, a molecule of AST comprises oxygenated ionone rings at both ends and a conjugated polyene chain linking them, forming a unique polar–nonpolar–polar structure. The hydroxyl (-OH) and carbonyl (-CHO) groups contained in the flanking rings and the high unsaturation in their structure contribute to the excellent antioxidant activity of AST. Studies have shown that AST exerts its protective effect mainly by enhancing transmembrane electron transport, quenching ROS, and upregulating the anti-oxidative stress pathway Nrf2/Keap1 [18,19].

Natural AST is commonly found in aquatic animals such as salmon, shrimp, and crustaceans, while the most abundant source is the microalgae species *Haematococcus pluvialis* [20]. In the wild, AST can be synthesized de novo in a wide variety of bacteria, yeasts, microalgae, protists, and a limited variety of plants (such as *Adonis aestivalis*) and subsequently enriched in aquatic animals through the food chain [21]. The precursors for AST synthesis in *Haematococcus pluvialis* are mainly derived from β-carotene, which is catalyzed by β-carotene ketolase and hydroxylase to generate keratin and zeaxanthin metabolites, respectively [22].

The health benefits of AST have been well documented by many studies. AST has been shown to enhance immunity, prevent cardiovascular diseases, protect the nervous system, improve skin condition, attenuate diabetes, etc. [23]. Brendler and Williamson reviewed the safety issues of AST, which did not elicit serious adverse reactions even at very high doses (i.e., 45 mg per day, which is about twice the maximum recommended daily dose) [24]. Thus, AST has gained widespread attention and application globally. The global market size of AST exceeded $110 million in 2018 and is growing annually [24]. In the past decade, the research on AST in health has grown exponentially, and increasing evidence has proven the beneficial effect of AST on MS. In 2022, a meta-analysis of seven randomized controlled trials by Leung et al. showed AST dietary supplementation has a significant attenuating effect on dyslipidemia [25]. Radice et al. reviewed the effects of AST on animal models of obesity-associated diseases in 2021 and indicated that AST had notable lipid-lowering and hypoglycemic bioactivities [26]. Abbasian et al. summarized the function of carotenoids in improving hypertension and indicated that AST had antihypertensive properties [27].

Currently, there is a lack of systematic review regarding the effects and mechanisms of AST on MS. Furthermore, the complexity of AST’s structure leads to a diversity of mechanisms through which it may ameliorate MS. Therefore, this article provides a comprehensive review of the basic properties of AST, the property differences imparted by its isomers and derivatives, and their ameliorative effects and mechanisms on the primary symptoms of MS.

## 2. Structure and Isomers of AST

The unique architecture of AST molecules gives rise to optical and geometric isomers. *E*ach β-ionone ring harbors two chiral carbon centers at the C-3 and C-3′ positions, yielding two enantiomers [(3S,3′S), (3R,3′R)] and one *meso* form (3R,3′S) [28]. Notably, the different enantiomeric forms of AST, analogous to AST, depend on how the hydroxyl (-OH) groups attach to these asymmetric carbon centers. Specifically, the “R-configuration” carbon atoms typically reside above the molecular plane, whereas their “S-configuration” counterparts lie below [23,29]. The presence of multiple conjugated double bonds in AST molecules, each occurring in either *Z* or *E* form, further contributes to the myriads of geometric isomers. In theory, there exist 271 *Z*-isomers, along with a single all-*E* isomer [23]. Commonly encountered isomers include the 9-*Z*, 13-*Z*, 15-*Z*, 9,13-*di*-*Z*, and 9,15-*di*-*Z* isomers [30]. However, due to the increased steric hindrance associated with *Z*-double bonds, *Z*-isomers exhibit weaker stability compared to the all-*E* isomer. Consequently, all-*E* AST dominates in natural occurrences. The hydroxyl groups located on the two ionone rings of AST can undergo esterification with various fatty acids, resulting in the formation of AST esters [31]. Research has demonstrated that AST esters exhibit distinct differences in stability and bioavailability compared to free AST. Specifically, free-form AST is highly unstable and susceptible to oxidation, often existing in the form synthesized through chemical processes. In contrast, esterified AST exhibits greater stability arising from the formation of esters between fatty acids and the hydroxyl groups present in each of its terminal cyclic structures. This form is more prevalent in nature. Furthermore, due to its enhanced lipid solubility, esterified AST is more readily absorbed by the human body. Consequently, esterified AST demonstrates a higher bioavailability within the body [31,32]. Furthermore, the C-C bonds between C-2 and C-3, as well as between C-2′ and C-3′ in the AST molecule, are capable of undergoing dehydrogenation to form C=C double bonds. Depending on the degree of dehydrogenation, AST can transform into semi-astacene and astacene, further diversifying its chemical profile [33,34].

## 3. In Vivo Absorption, Transport, and Metabolic Pathways of AST

**Figure 2:**
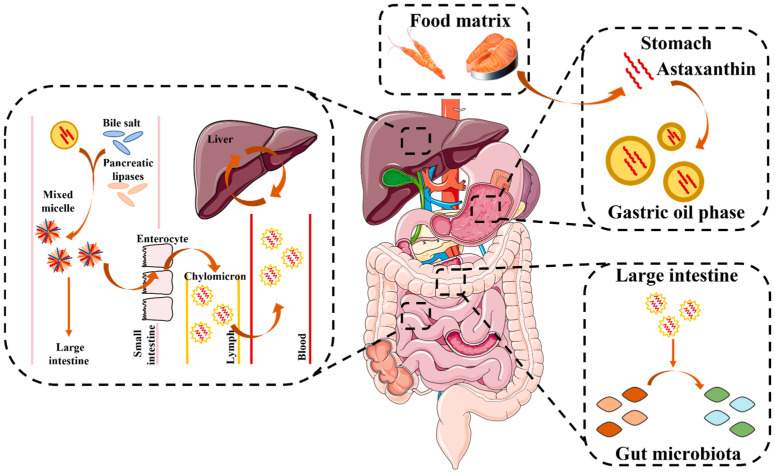
In vivo absorption, transport, and metabolism of free-form AST.

As shown in Figure 2, taking free-form astaxanthin as an example, similar to other lipid-soluble carotenoids, AST is digested and absorbed in a complex manner, including release from the food matrix, transfer to the stomach, formation of mixed micelles under the dissolution of pancreatic lipase and bile salts, transport through microvilli, uptake by intestinal epithelial cells, incorporation into chylomicrons, entry into the lymphatic system and circulation, and transport to the liver [34,35]. Absorption of carotenoids from the intestine (mainly in the duodenum) into enterocytes is primarily a passive process, and the percentage of the ingested dose that is transferred into micelles is referred to as bioaccessibility [36]. However, the bioaccessibility of orally administered AST is not ideal and is limited by poor water solubility and chemical stability [37].

The bioavailability of AST is partly related to its structure. In a clinical trial of healthy adult men, Østerlie et al. found that a single oral dose of 100 mg of mixed isomers treatment in healthy middle-aged male volunteers produced higher plasma concentrations of *Z*-AST, especially 13-*Z*-isomers [38], which is consistent with results from in vitro models and rat studies [39,40]. However, there was no significant difference in the pharmacokinetics of the AST stereoisomers [41]. Within the intestinal tract, esterified AST undergoes de-esterification to convert into free AST and fatty acids. The presence of these fatty acids facilitates the formation of mixed micelles with astaxanthin, enhancing its absorption by intestinal epithelial cells. Consequently, esterified AST exhibits a higher bioavailability compared to its free form. However, due to this mechanism, esterified AST typically does not reside in chylomicrons or human serum [32]. Although the specific enzymes that hydrolyze AST esters are unknown, lipases from the human pancreas have been proposed, and cholesterol esterases have also been used as digestive enzymes for de-esterifying AST esters in vitro [42]. Rao et al. separated AST extracted from *Haematococcus pluvialis* into free AST, monoester, and diester and gave them to rats at 200 μg/kg BW [43]. The contents of AST in serum and liver were the highest in the diester treatment group, followed by the single-ester treatment group, suggesting that AST ester had better bioavailability than free AST. Yang et al. synthesized AST esters with different fatty acids in the laboratory and evaluated their stability and bioavailability using in vitro and in vivo digestion models [44]. The antioxidant studies showed that the DPPH and ABTS scavenging rates of Asta-C18:0, Asta-C18:1, Asta-C18:2, and Asta-C22:6 increased gradually. Among all Asta-*E*s, Asta-C22:6/C22:6 showed the highest antioxidant capacity. According to reports, the stability and bioavailability of astaxanthin esters are influenced by the length and degree of unsaturation of the carbon chain, yet this relationship is not strictly positive [45]. However, a consensus has emerged that esterified astaxanthin, on the whole, exhibits superior stability and bioavailability compared to free astaxanthin.

AST, due to its poor water solubility and instability, is prone to oxidation, resulting in limited bioavailability upon direct ingestion. Today, many delivery systems, such as emulsions, nanoparticles, and liposomes, have been designed to enhance the stability and bioavailability of AST [46,47,48]. Xu et al. successfully encapsulated AST within gelatinized octenyl succinic anhydride (OSA)-modified starch, significantly enhancing the retention rate and long-term stability of AST [49]. Furthermore, in vitro digestion experiments demonstrated that the OSA starch exhibited excellent protective properties towards AST. *Z*heng et al., on the other hand, utilized Pickering emulsions (P*E*s) for AST encapsulation, finding that a P*E* with a mixed oil phase of olive oil and edible tea oil achieved the optimal encapsulation effect [50]. Following simulated gastrointestinal digestion, this formulation exhibited the highest retention rate and bioaccessibility of AST. Odeberg et al. prepared lipid formulations containing different synthetic surfactants to promote bile salt secretion, lipase activity, and coeliac formation in humans [51]. It promotes more AST to be encapsulated in chyle, thereby enhancing the absorption of AST in the small intestine. Tachaprutinuna et al. successfully developed microcapsules encapsulating AST using chitosan as the raw material and significantly enhanced AST’s dispersion capability in water and thermal stability [52]. Liu et al. encased free-form AST within poly (lactic-co-glycolic acid) (PLGA) nanoparticles and subsequently coated them with chitosan to form microcapsules [53]. This innovative strategy not only improved the stability and water dispersibility of AST but also augmented the cellular compatibility of the nanoparticles. Different materials used for loading or encapsulating AST can provide varying degrees of protection to AST and offer potential for targeted delivery. In the future, the targeted delivery of AST holds promising application prospects in various fields, particularly in medical health and functional foods. More effective delivery media can reduce the in vivo diffusion and spontaneous oxidative decomposition of AST, thereby transporting a greater amount of active ingredients to the target organs. There is a pressing need for more research and development to create efficient and safe AST delivery systems, which will provide support for the production and application of AST.

## 4. AST and Obesity

Obesity is a pivotal element and the foremost diagnostic indicator of MS. Currently, obesity has become a global health concern. According to the World Obesity Report 2024 [54], the number of adults affected by overweight/obesity is projected to surge from 2.2 billion in 2020 to 3.3 billion by 2035, with the global prevalence escalating from 42% to 54%. This epidemic trend will lead to a significantly increased risk of metabolic disorders like hyperlipidemia, cardiovascular diseases, hypertension, and type II diabetes. As shown in Table 1, plenty of animal studies suggest that AST can ameliorate obesity. Chen et al. demonstrated that incorporating 0.02% AST into the high-fat diet (HFD) of mice for 16 weeks reduced their weight gain by 16.8% and fat mass by 24.0% [55]. Similarly, Wang et al. observed a dose-dependent decrease in weight gain and adipose tissue weight in HFD-fed mice supplemented with 0.25%, 0.5%, or 0.75% AST in daily diets over a 9-week period, with the 0.75% AST group exhibiting a reduction of approximately 30% [56]. Radice et al. conducted a meta-analysis of 17 animal studies encompassing three distinct models: MS, type 2 diabetes mellitus (T2DM), and nonalcoholic fatty liver disease (NAFLD) [26]. The meta-analysis revealed that AST exhibited a significant capability to reduce adipose tissue mass across all three animal models.

However, the use of AST has shown mixed results in attenuating obesity in humans. Limited clinical evidence suggests that AST can lead to reductions in weight or BMI. A double-blind, randomized controlled trial (RCT) conducted by Heidari et al. in 2023, involving 50 patients with Coronary Artery Disease (CAD), demonstrated that a daily intake of 12 mg of AST over an 8-week period significantly reduced the participants’ BMI, as well as their waist and hip circumferences [57]. Additionally, a population-based study by Saeidi et al. in 2023, encompassing 68 obese male subjects, showed that a daily dose of 20 mg of AST administered for 12 weeks effectively decreased the BMI of the participants [58]. In contrast, Xia et al. conducted a meta-analysis encompassing 13 double-blind, randomized controlled trials (RCTs) spanning from 2007 to 2018, involving a diverse range of participants, including healthy individuals, patients with type 2 diabetes mellitus (T2DM), and obese individuals [59]. Their analysis revealed that AST intervention did not significantly impact the weight or body mass index (BMI) of the participants. In another meta-analysis involving seven double-blind RCTs, Leung et al. also concluded that AST intervention had no notable effect on BMI among healthy individuals, T2DM patients, and obese populations [25]. This discrepancy between animal and human studies could be attributed to differences in dosage. The majority of doses utilized in human studies were below 20 mg/day [25], which equals approximately 3.3 mg/kg/day in mice. Conversely, animal experiments typically employed doses of AST around 30 mg/kg/day or higher [26], dramatically higher than those administered to humans. Therefore, future human trials with higher doses of AST are necessary to validate the anti-obesity efficacy of AST in the general or obese population. It is noteworthy that the AST used in the aforementioned experiments did not distinguish between structures and did not account for potential differences in effects and mechanisms that may arise from variations in AST’s structure.

The exact mechanisms by which AST improves obesity are unclear, although several potential mechanisms were raised. AST primarily modulates HFD-induced obesity via modulating the adenosine monophosphate-activated protein kinase (AMPK)/sterol regulatory element-binding protein 1c (SREBP1c) pathway. AMPK has been identified as a pivotal regulator in maintaining cellular energy homeostasis, regulating various metabolic and physiological processes related to lipid degradation [60]. SREBP1c, a transcription factor managing lipogenesis, undergoes significant downregulation upon AST supplementation in mice [61]. AST exerts an activating effect on AMPK in human white adipocytes, which in turn upregulates the expression of peroxisome proliferator-activated receptor alpha (PPARα) [62]. PPARα, in conjunction with peroxisome proliferator-activated receptor gamma coactivator-1 alpha (PGC-1α), plays a pivotal role in regulating the homeostasis of adipose tissue by jointly modulating the balance between fatty acid synthesis and oxidation. The interplay between PPARα and PGC-1α promotes fatty acid oxidation and, to a certain extent, inhibits the expression of SREBP1c [61,62]. Furthermore, AST reduces the expression of downstream targets related to lipid and cholesterol synthesis, such as fatty acid synthase (FAS) and Stearyl CoA Desaturase 1 (SCD-1), while upregulating genes involved in lipid oxidation, including AdipoR1, PGC-1α, PPARα, and LXRα in Penaeus monodon [63]. Secondly, the composition and metabolic functions of the gut microbiota are recognized to influence the development of obesity [64]. Wang et al. conducted an analysis of gut microbiota in mice fed an HFD with and without AST intervention, revealing that AST restored obesity-related intestinal flora dysbiosis by increasing the abundance of gut bacteria essential for weight loss (e.g., *Bacteroidetes* and *Verrucomicrobia*) and decreasing those of pathogenic microbiota (e.g., *Firmicutes* and *Proteobacteria*) [61]. Furthermore, evidence suggests that short-chain fatty acids (SCFAs) produced by intestinal microbiota metabolism can inhibit obesity [65]. A study conducted by Zhang et al. found that AST intervention significantly increased the levels of SCFAs, such as acetate and propionate, in the feces of mice [66]. Therefore, AST may also alleviate obesity by improving the metabolites of intestinal microbiota. Thirdly, AST might relieve obesity by maintaining mitochondrial homeostasis. Accumulating evidence has underscored mitochondrial fission as a crucial process for maintaining metabolic homeostasis, with its dysregulation implicated in various metabolic disorders like obesity [67]. Nishida et al. reviewed the molecular targets of AST and indicated that AST improved mitochondrial stability and enhanced mitochondrial function through activation of the AMPK/Sirtuins (SIRTs)/PGC-1 pathway in mice [68]. In addition, AMPK controls mitochondrial quality by facilitating mitochondrial biogenesis, regulating mitochondrial fission and fusion via phosphorylation of Mef, and inducing mitophagy through phosphorylation of Ulk-1 in damaged mitochondria [69,70,71]. Thereby, AST’s activation of AMPK is intimately linked to its regulatory effects on metabolic homeostasis. Furthermore, AST has been implicated in enhancing muscle function and facilitating lipid metabolism during exercise, which contributes to reduced body fat in mice [72]. AST was also reported to reduce subcutaneous fat accumulation by elevating the circulating levels of Human High-Molecular Weight Adiponectin (HMW-ADI), which inhibits the development of obesity by regulating fat metabolism and energy balance [73].

**Table 1:** Summary of the effects of AST on obesity.

Study	Model	Dose of AST	Duration of Intervention	Outcomes	Mechanism
**ANIMALS**					
Chen Y., 2023 [55]	C57BL/10ScSnDmdmdx/J mice fed with HFD	0.02%	16 weeks	BW ↓ Body weight gain ↓ Weight of subcutaneous fat masses and epididymal fat masses ↓	*Akkermansia*, *Bifidobacterium*, and *Butyricicoccus* ↑
Wang X., 2022 [74]	C57BL/6J mice fed with HFD	50 mg/kg/day	8 weeks	Body weight gain ↓	mRNA levels of LC3A/B, ATG5, ATG7, and Beclin1 ↑
Shatoor A., 2022 [75]	Wistar rats fed with HFD	6 mg/kg/day	8 weeks	Final body weight ↓	miR-21 ↓ Nrf2 ↑
Li Y., 2022 [76]	ICR mice fed with HFD	60 mg/kg/day	10 weeks	BW ↓ Epididymal fat index ↓	Nrf2 nuclear translocation ↑ AMPK signaling pathway ↑ SREBP-1, FAS, and ACC signaling pathways ↓
Wang M., 2021 [61]	C57BL/6 mice fed with HFD	0.25%, 0.5%, 0.75%	9 weeks	Body weight gain ↓ 0.75% AST group: Visceral adipose tissue ↓	AMPK, SREBP1c, ACC, FAS, PPARγ, and SCD-1 ↓ PPARα ↑ F/B ↓ *Allobaculum* ↑ *Akkermansia* ↑
Nishida Y., 2020 [69]	C57BL/6J mice fed with HFD	0.02%	8, 16, 24 weeks	BW ↓	Mitochondrial oxidative phosphorylation and FFA metabolism ↑ AMPK-PGC-1α pathway ↑
Chen Y., 2020 [77]	C57BL/KsJ mice db/db mice	30 mg/kg/day	3 weeks	BW ↓	Nrf2/HO-1 signaling pathway ↑
Wu L., 2020 [78]	C57 mice fed with HFD	10, 30, 60 mg/kg/day	12 weeks	Body weight gain ↓	FGF21, FOXA, and PGC-1α, PPARα ↑ CD36, FATP5, SREBP-1c ↓
Kim B., 2017 [79]	C57BL/6J mice fed with HF/HS	0.03%	30 weeks	BW ↓ eWAT ↓	CD11c and MCP-1 ↓ CPT-1α, ACOX-1, and PPARα ↑
Jia Y., 2016 [80]	C57BL/6J mice fed with HFD	6, 30 mg/kg/day	8 weeks	eWAT ↓	PPARγ, Akt, and SREBP1 ↓ PPARα ↑
Arunkumar E., 2012 [81]	Mus musculus albino mice	6 mg/kg/day	8 weeks	BW ↓ eWAT ↓	IRS-1-associated PI3-kinase expression, p-Akt/Akt ratio, and GLUT-4 translocation ↑
Bhuvaneswari S., 2010 [82]	Mus musculus albino mice	6 mg/kg/day	8 weeks	BW ↓	CYP2E1 activity, TGF-1 expression, and MPO activity ↓
Ikeuchi M., 2007 [83]	Obese mice fed with HFD	1.2, 6, 30 mg/kg/day	60 days	Body weight gain ↓	/
**CLINICAL TRIALS**					
Heidari M., 2023 [57]	50 CAD patients	12 mg/day	8 weeks	BMI ↓ Hip circumference (HC) ↓ Waist circumference (WC) ↓	/
Saeidi A., 2023 [58]	68 obese males	20 mg/day	12 weeks	BMI ↓	/
Liu S., 2018 [84]	42 old subjects	12 mg/day	4 months	Body weight- BMI -	/
Mashhadi N., 2018 [85]	44 T2DM patients	8 mg/day	8 weeks	Body weight- BMI -	/
Chen J., 2017 [86]	29 healthy climacteric women	12 mg/day	3 months	Body weight- BMI -	/
Canas J., 2017 [73]	20 obese human kids	500 μg×2/day	6 months	BMI z-score ↓ Waist-to-height ratio ↓ Subcutaneous adipose tissue ↓	Circulating concentrations of HMW-ADI ↑
Coombes J., 2016 [87]	61 renal transplant patients	12 mg/day	1 year	Body weight- BMI -	/
Baralic I., 2016 [88]	40 trained male soccer players	4 mg/day	90 days	Body weight- BMI -	/
Choi H., 2011 [89]	23 obese patients	5, 20 mg/day	3 weeks	BMI -	/

↑: Up-regulated; ↓: Down-regulated; -: Have no significant impact.

## 5. AST and Hyperglycemia

Hyperglycemia is another critical pathological feature of MS that is characterized by elevated blood glucose levels resulting from insufficient insulin secretion or insulin resistance (IR). Insulin resistance (IR) is a state of declined sensitivity of insulin receptors within the body, leading to a diminished hypoglycemic effect of insulin [90]. IR is recognized as the pathological core disorder of MS [91] and is closely associated with an increased risk of serious metabolic diseases, like cardiovascular diseases and type 2 diabetes mellitus (T2DM) [92,93,94]. As presented in Table 2, there is evidence indicating that AST is associated with the regulation of hyperglycemia and IR.

In the study conducted by Liao et al., largemouth bass were fed a high-carbohydrate diet supplemented with 0.01% AST for a duration of 8 weeks [95]. Notably, the serum glucose levels of the largemouth bass decreased significantly while the serum insulin levels increased markedly. Additionally, during the glucose tolerance test (GTT), a significant reduction was observed in the area under the curve (AUC) of the GTT profile. Gowd et al. have reviewed the therapeutic efficacy of AST in diabetic animal models, wherein AST consistently displayed favorable outcomes in 11 studies and effectively reduced postprandial blood glucose levels and the Homeostasis Model Assessment of Insulin Resistance (HOMA-IR) index [96]. AST’s hypoglycemic effects have also been validated in human IR or diabetic patients. Jabarpour et al. conducted a clinical trial involving 58 patients with polycystic ovarian syndrome, administering 12 mg/day of AST for 8 weeks [97]. This study revealed a notable reduction in fasting blood glucose levels, fasting insulin levels, Quantitative Insulin Sensitivity Check Index (QUICKI), and HOMA-IR indices among participants. Similarly, Saeidi et al.’s experiment demonstrated that a daily intake of 20 mg AST over 12 weeks, in conjunction with high-intensity exercise, effectively decreased blood glucose levels, blood insulin levels, and HOMA-IR indices in obese male patients [58]. However, a meta-analysis conducted by Leung et al. in 2022, encompassing seven double-blind, randomized controlled trials (RCTs), pointed out that AST did not significantly reduce fasting blood glucose levels in healthy individuals or patients with type 2 diabetes mellitus (T2DM) [25]. This is in contrast to the results of the above two experiments, suggesting that differences in patient populations may underlie the varying efficacy of AST. More clinical trials are necessary to reveal the effects of AST on hyperglycemia in humans. The discrimination of AST isoforms was also neglected in the above experiments.

**Figure 3:**
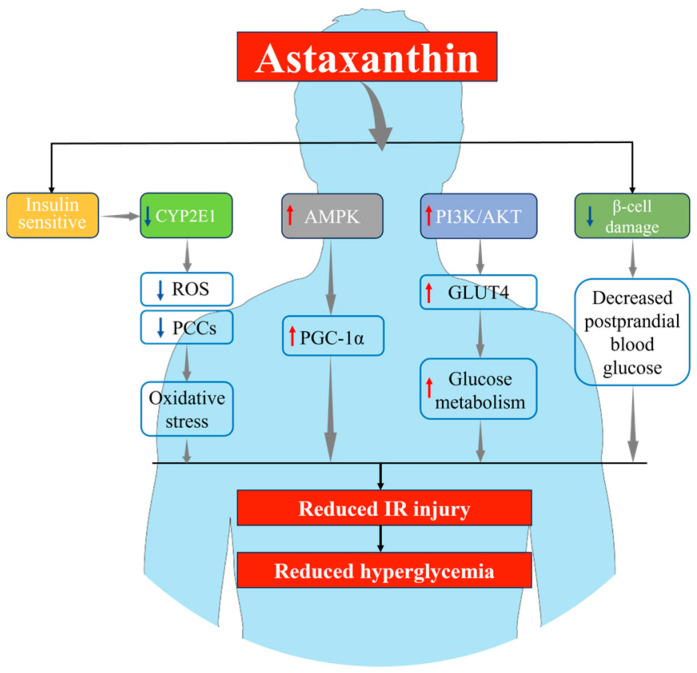
Mechanism diagram of AST alleviating IR.

As shown in Figure 3, numerous studies explained the potential mechanisms underlying the protective effects of AST on hyperglycemia. Cytochrome P450-2e1 (CYP2E1), a member of the cytochrome P450 family ubiquitous in hepatic cells, acts as a pro-oxidant enzyme that is inhibited by insulin [98]. Consequently, IR or reduced insulin sensitivity may lead to the upregulation of CYP2E1, contributing to hepatic damage under IR conditions through the excessive generation of reactive oxygen species (ROS). Studies have shown that AST significantly improves insulin sensitivity and reduces hepatic CYP2E1 expression in mice, thereby inhibiting hepatic IR and the associated liver injury [82]. Huang et al. elucidated that insulin binding to insulin receptors in skeletal muscle cells activates the PI3K/Akt signaling pathway, ultimately leading to the translocation of glucose transporter 4 (GLUT4) from intracellular compartments to the plasma membrane, enhancing glucose uptake and metabolism [99]. Arunkumar et al. confirmed that AST increases the phosphorylated Akt/Akt ratio and GLUT-4 translocation in the skeletal muscle of HFD mice to activate PI3K/Akt signaling pathway transduction and inhibit IR in mice [81]. Additionally, AMPK promotes glucose uptake and fatty acid oxidation in skeletal muscle. Similarly, Liao et al. demonstrated that activation of the PI3K/Akt pathway occurred in the livers of largemouth bass [95]. By activating the PI3K/Akt pathway, AST inhibited the anti-apoptotic activity induced by a high-sugar diet and significantly improved hepatic insulin resistance [95]. Beyond upregulating GLUT4 expression, PGC-1α plays a pivotal role in AMPK’s anti-IR activity. AMPK, serving as an energy sensor, regulates the expression and activity of PGC-1α. In turn, PGC-1α promotes the expression and membrane translocation of GLUT4 by modulating multiple transcription factors and signaling pathways. GLUT4, as a glucose transporter located on the cell membrane, plays a crucial role in influencing cellular glucose uptake and utilization. These regulatory interactions collectively maintain cellular energy, metabolic homeostasis, and insulin sensitivity [100]. Nishida et al. demonstrated that AST enhances glucose uptake in skeletal muscle cells (C2C12) and inhibits IR via the activation of the AMPK-PGC-1α signaling pathway in mice [69]. Furthermore, AST protects β-cell function, thereby hindering the progression from IR to diabetes. The protective role of AST on β-cells was initially observed in 2002 when Uchiyama et al. administered 1 mg/day of AST to C57BL/KsJ-db/db mice for 18 weeks and found that AST significantly lowered their postprandial blood glucose and mitigated β-cell damage [101]. Adipose tissue is another crucial target for insulin. In adipose tissue, insulin’s primary role is to stimulate glucose uptake and inhibit lipolysis. It has been reported that during IR, impaired inhibition of lipolysis is associated with glucose intolerance and elevated plasma free fatty acid (FFA) levels [102]. These events have also been shown to block insulin signaling in skeletal muscle, amplify hepatic gluconeogenesis, and disrupt glucose-stimulated insulin responses. Furthermore, studies have indicated that chronic low-grade inflammation accompanies fat accumulation in adipose tissue, suggesting that obesity-related chronic inflammation plays a role in the development of IR [81]. The accumulation of FFAs in adipose tissue may induce pro-inflammatory and insulin-desensitizing effects through Toll-like receptor-mediated pathways, thereby adversely affecting the insulin signaling cascade that contributes to IR. Ranade et al. reported that administering 24 mg/kg/day of AST to Wistar rats for a duration of 6 weeks resulted in the downregulation of fatty acid synthase (FASN), fructose-bisphosphate aldolase A (AldoA), and pyruvate kinase isoform M2 (PKM2) in adipose tissue [103]. This treatment also inhibited the synthesis of methylglyoxal (MG), thereby maintaining glycolytic stability and enhancing insulin sensitivity, ultimately suppressing IR. In both humans [85] and rodents [104], AST can increase blood levels of adiponectin, which is a well-known insulin-sensitizing hormone. In brief, AST primarily controls the occurrence of hyperglycemia by inhibiting IR, and this protective effect is not confined to a specific tissue or organ; rather, it is systemic in nature.

**Table 2:** Summary of the effects of AST on hyperglycemia.

Study	Model	Dose of AST	Duration of Intervention	Outcomes	Mechanism
**ANIMALS**					
Liao Z., 2024 [95]	Largemouth bass fed a high-carbohydrate diet	0.01%	8 weeks	Serum glucose, AUC of GTT ↓ Serum insulin ↑	PI3K/Akt pathway, AMPK pathway, GLUT4, PGC-1α ↑
Nishida Y., 2020 [69]	C57BL/6J mice fed an HFD	0.02%	8, 16, 24 weeks	Blood glucose, blood insulin ↓ Insulin sensitivity ↑	AMPK activation ↑ Mitochondrial biogenesis ↑
Gao Y., 2020 [105]	C57BL/6J mice fed an HFD	50 mg/kg/day	8 weeks	Blood glucose, blood insulin ↓ HOMA-IR ↓	*Verrucomicrobia*, *Akkermansia* ↓ *Bacteroides,* *Coprococcus* ↑
Chen Y., 2020 [77]	C57BL/KsJ db/+ mice	30 mg/kg/day	20 days	Blood glucose, blood insulin ↓	Function of the pancreatic β-cells ↑ Nrf2/HO-1 signaling pathway ↑
Kim B., 2017 [79]	C57BL/6J mice fed an HF/HS	0.03%	30 weeks	Blood glucose ↓	/
Ni Y., 2015 [106]	Male C57BL/6 mice and male ob/ob mice	0.02%	10 weeks	Blood glucose ↓ Insulin sensitivity ↑	Srebp1c, CD36, p38, MAPK, NF-κB p65, TGF-β1, Cd11c, MCP-1 ↓ p-Akt/Akt, CD163 ↑
Bhuvaneswari S., 2012 [107]	Adult male Mus musculus mice	2 mg/kg/day	45 days	Blood glucose, blood insulin ↓ HOMA-IR ↓	Tyrosine phosphorylation ↑ Serine phosphorylation of insulin receptor substrates (IRS)-1 and -2 ↓ IRS–PI3K–Akt pathway ↑ JNK-1 and ERK-1 activity ↓
Arunkumar E., 2012 [81]	Adult male Mus musculus albino mice	6 mg/kg/day	60 days	Blood glucose, blood insulin ↓	Tyrosine phosphorylation of IR-β ↑ GLUT-4 protein level ↑
Hussein G., 2007 [108]	Male SHR/NDmrc-cp rats	50 mg/kg/day	18 weeks	Blood glucose ↓ HOMA-IR ↓	/
Uchiyama K., 2002 [101]	Female db/db mice	1 mg/day	18 weeks	Blood glucose ↓	Function of the pancreatic β-cells ↑
**CLINICAL TRIALS**					
Ciaraldi T., 2023 [37]	Adults with dyslipidemia and prediabetes	12 mg/day		Insulin action CVD risk markers ↓	/
Urakaze M., 2021 [109]	53 healthy subjects	12 mg/day	12 weeks	Blood glucose, blood insulin ↓	HbA1c ↓
Mashhadi N., 2018 [85]	Participants with definitive diagnosis of type 2 diabetes with no insulin therapy	8 mg/day	8 weeks	Glucose concentrations ↓	Blood adiponectin concentration ↑

↑: Up-regulated; ↓: Down-regulated.

## 6. AST and Dyslipidemia

Dyslipidemia, a pivotal component of MS, is characterized by elevated levels of triglycerides (TG), total cholesterol (TC), and low-density lipoprotein cholesterol (LDL-C), as well as decreased levels of high-density lipoprotein cholesterol (HDL-C) in plasma. Table 3 presents the relevant article information on AST mitigating dyslipidemia. Wang et al. demonstrated that the addition of 0.5% or 0.75% free-form AST to an HFD for 9 consecutive weeks markedly reduced serum TC and TG levels in mice fed an HFD. However, there was no significant effect on LDL-C and HDL-C [56]. Ni et al. reported that the addition of 0.02% AST to a cholate diet (CL) for 12 weeks markedly reduced plasma TC, TG, and non-esterified fatty acid (NEFA) levels in mice, and the effect is more effective than vitamin E [106]. Wu et al. further showed that administering 5, 15, or 30 mg/kg/day AST to hyperlipidemia mice induced by HFD feeding for 10 weeks resulted in a dose-dependent decline in serum TC and TG levels [78]. The changes in LDL-C and HDL-C were not reflected. Dyslipidemia disrupts lipid metabolism homeostasis within the body, often accompanied by excess lipid accumulation in organs, such as hepatic lipid deposition and NAFLD [110]. A review by Sayuti et al. in 2023 summarized the effects of AST on dyslipidemia in NAFLD animal models, wherein AST significantly reduced serum TC, TG, and LDL-C levels in mice across 11 studies included [111]. However, the impact of AST on serum HDL-C levels appeared inconsistent based on the review. Additionally, in these experiments, AST reduced the levels of TC and TG in the liver. However, evidence for its regulation of LDL-C and HDL-C in the liver was lacking.

Recently, the effects of AST on dyslipidemia in human subjects have also been validated. Ciaraldi et al. conducted a clinical trial involving 36 male and female volunteers with prediabetes and dyslipidemia who received 12 mg of AST daily for 24 weeks [37]. The results indicated a significant reduction in serum TC and LDL-C levels, with no notable effects on HDL-C or TG levels. In contrast, Jabarpour et al.’s clinical trial observed a decrease in serum LDL-C levels and an increase in HDL-C levels after 12 mg/day AST intervention lasted for 8 weeks, while the impact on TC and TG was insignificant [97]. Urakaze et al. performed a clinical trial encompassing 53 subjects, demonstrating that 12 weeks of 12 mg/day AST intake significantly reduced plasma LDL-c levels yet did not significantly affect TC, TG, or HDL-C levels [109].

The modulation of lipid metabolism and improvement of dyslipidemia by AST are complicated and linked to its regulation of oxidative stress and metabolic pathways. Dietary lipids, primarily triglycerides and cholesterol, are absorbed in the small intestine and transported to the liver for metabolism [112]. This process involves initial lipolysis by pancreatic lipases in the small intestine, emulsification by bile to form chylomicrons, their transportation through the intestinal lymphatic system into the bloodstream, and subsequent uptake and metabolism by hepatocytes [112]. Thus, the liver plays a pivotal role in regulating lipid homeostasis.

**Figure 4:**
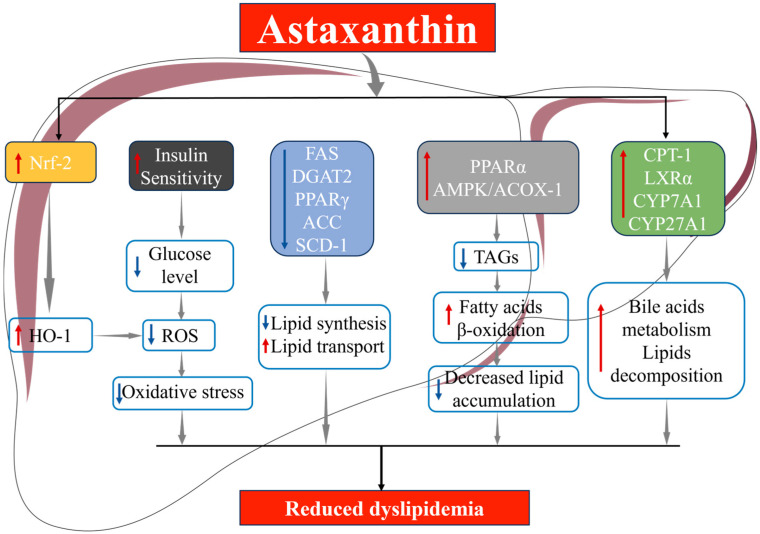
Mechanism diagram of AST alleviating dyslipidemia.

As shown in Figure 4, AST can ameliorate oxidative stress by reducing blood glucose levels and consequently decreasing ROS. Furthermore, studies have demonstrated that AST can further inhibit ROS production and alleviate subsequent liver injury and dyslipidemia through activation of the Nrf2/HO-1 signaling pathway in mice [77]. Wang et al. reported that AST treatment significantly reduced expression levels of lipid synthesis-related genes, like acetyl-CoA carboxylase (ACC), Fas cell surface death receptor (FAS), and stearoyl-CoA desaturase 1 (SCD-1) in the liver of HFD fed mice, and upregulated those genes associated with lipid oxidation and bile acid metabolism, such as carnitine palmitoyltransferase-1 (CPT-1), liver X receptor α (LXRα), cholesterol 7α-hydroxylase 1 (CYP7A1), and mitochondrial sterol 27-hydroxylase (CYP27A1) [56]. Furthermore, extensive evidence suggests that gut microbiota and their metabolites contribute to the host’s lipid metabolic balance [113]. Pratap et al. found that 0.5 mg/day AST administration to normal-diet-fed mice significantly altered gut microbiota composition, with increased abundance of various probiotic bacteria, like *Bacteroidia*, *Bacilli*, *Clostridia*, and *Verrucomicrobia* [114]. Many of these microbial species have been reported to produce short-chain fatty acids (SCFAs) and other metabolites in the gut, contributing to maintaining intestinal barrier integrity and improving dyslipidemia [115,116]. Wang et al. observed that 0.75% AST dietary supplementation to HFD-fed mice for 9 weeks significantly decreased the fecal abundance of Firmicutes and Proteobacteria while increasing that of Bacteroidetes and *Verrucomicrobia*, *Allobaculum*, and *Akkermansia* [56]. The genus of *Allobaculum* is associated with hormone secretion, SCFA production, serum HDL-C concentrations, and intestinal barrier integrity [117,118]. *Akkermansia*, a mucosal-associated gut commensal, is recognized as a probiotic bacteria and intimately linked to adiposity, secondary bile acid biosynthesis, and IR [119]. Muscle and adipose tissue are also closely involved in maintaining lipid metabolic balance [120]. Nishida et al. elucidated that AST activated AMPK in skeletal muscle and upregulates the expression of transcription factors Mef and Ulk-1 in mice [69], thereby inducing mitochondrial remodeling and enhancing fatty acid oxidative phosphorylation and β-oxidation [105]. This finding is also supported by Yang et al., who emphasized AST’s lipid-lowering mechanism by inducing the transcription of acyl-CoA oxidase 1 (ACOX-1), a crucial enzyme responsible for fatty acid oxidation in mice [121]. Moreover, AST was found to regulate the expression of peroxisome proliferator-activated receptors (PPARs) [121], a nuclear receptor subfamily that controls various target genes involved in lipid metabolism and glucose homeostasis [122]. To be specific, AST upregulates PPARα and downregulates PPARγ. Kobori et al. concurred, stating that AST improves fatty acid transport, metabolism, and oxidation by increasing PPARα transcription, thus reducing their accumulation in adipocytes of mice [123]. In addition, AST can enhance energy metabolism and regulate blood lipids in T2DM patients by upregulating the SIRT1-AMPK pathway in peripheral blood mononuclear cells (PBMCs) [124].

**Table 3:** Summary of the effects of AST on dyslipidemia.

Study	Model	Dose of AST	Duration of Intervention	Outcomes	Mechanism
**ANIMALS**					
Nguyen-Le D., 2023 [125]	Female Swiss mice	30 mg/kg/day	16 weeks	Plasma: TC, TG, LDL-c ↓ Maintaining blood cholesterol	/
Wang M., 2022 [56]	Male C57BL/6 mice	0.25%, 0.5%, 0.75%	9 weeks	Serum: TC, TG, LDL-c ↓ Liver: TC, TG ↓ Lipid metabolism ↑ Cholesterol metabolism ↑	CAT, SOD, GSH, CPT-1, Lxrα, CYP7A1, CYP27A1 ↑ ACC, FAS, SCD-1 ↓
Li Y., 2022 [76]	Male ICR mice fed an HFD	60 mg/kg/day	10 weeks	Liver: TC, TG, LDL-c ↓ HDL-c ↑ Serum: TC, TG, LDL-c ↓ HDL-c ↑	AMPK, nuclear-Nrf2 ↑ SREBP-1 ↓ *Eubacterium xylanophilum*, *Candidatus Saccharimonas*, *Lactococcus*, *Faecalibacterium*, and *Dubosiella* ↑
Wu L.,2020 [78]	Male C57BL/6 mice	10, 30, and 60 mg/kg/2 days	10 weeks	Serum TG ↓ Lipid decomposition ↑ Fatty acid oxidation ↑	FGF21/PGC-1α pathway, PPARα, OXPHOS ↑ Mitochondrial dysfunction, PPARγ ↓
Xu J., 2017 [126]	Male Sprague–Dawley rats	-	10 weeks	Liver: TC, TG ↓ Circular lipid droplets ↓	PPARα, CPT-1, ACO, SOD, CAT, GPx, GSH ↑ SREBP1, HMGCR, FAS, ACC, TBARs ↓
Kim B., 2017 [79]	C57BL/6J mice fed an HF/HS	0.03%	30 weeks	Liver: TC, TG ↓	CD11cm MCP-1 ↓ CPT-1α, ACOX-1, PPARα ↑
Ni Y., 2015 [106]	Male C57BL/6 mice and male ob/ob mice	0.02%	10 weeks	Plasma: TC, TG ↓ Lipid peroxidation ↓	Srebp1c, CD36, p38, MAPK, TGF-β1, Cd11c, MCP-1 ↓ p-Akt/Akt, CD163 ↑
Yang Y., 2014 [121]	Male C57BL/6 mice	0.003%, 0.01%, 0.03%	12 weeks	0.03% AST plasma: HDL-c ↑ TG ↓	ACOX-1, TGF-β1, Nrf2 ↑
**CLINICAL TRIALS**					
Jabarpour M., 2023 [97]	58 infertile females	2 × 6 mg/day	8 weeks	Serum: LDL-c, TC/HDL-c ↓ HDL-c ↑	/
Taghiyar S., 2023 [124]	60 T2DM patients	10 mg/day	12 weeks	Serum: Saturated fatty acids ↓ Arachidonic acid ↓ Polyunsaturated fatty acids ↓	SIRT1-AMPK pathway ↑
Ciaraldi T., 2023 [37]	36 men and women with prediabetes and dyslipidemia	12 mg/day	24 weeks	Plasma: TC, LDL-c ↓	/
Heidari1 M., 2023 [57]	50 CAD patients	12 mg/day	8 weeks	Serum: TC, LDL-c ↓	/
Urakaze M., 2021 [109]	53 healthy subjects	12 mg/day	12 weeks	Serum: Apolipoprotein E, MDA-LDL ↓	/
Landi F., 2019 [127]	50 dyslipidemia subjects	0.5 mg/day	6 weeks	Plasma: TG, LDL-c ↓	/
Maki K., 2015 [128]	102 dyslipidemia subjects	12 mg/day	8 weeks	Serum: TG, LDL-c ↓	/

↑: Up-regulated; ↓: Down-regulated.

## 7. AST and Hypertension

Hypertension is an essential criterion for diagnosing MS and is highly prevalent among MS patients. Hypertension is defined by systolic blood pressure (SBP) ≥ 140 mmHg and/or diastolic blood pressure (DBP) ≥ 90 mmHg [129]. In the PAMELA study, elevated blood pressure emerged as the most frequent symptom in MS patients, affecting over 80% of subjects [130]. The Global Cardiometabolic Risk Profile in Patients with Hypertension Disease (GOOD) study revealed that more than two-thirds of hypertensive patients have blood pressure levels within a high-risk range. The evidence for AST’s regulation of blood pressure is presented in Table 4.

In 2005, groundbreaking research was published highlighting the antihypertensive effects of AST. The study observed that AST significantly reduced blood pressure in both spontaneous hypertension rats (SHR) and stroke-prone SHR (SHR-sp) while also delaying the onset of stroke in these animal models [23]. Preuss et al. demonstrated that 100 mg/kg/day AST intake for eight months significantly reduced SBP and DBP in rats with hypertension induced by high sucrose intake and insulin resistance [131]. Similarly, Gao et al. employed Dahl salt-sensitive (S) rats fed a high-salt diet (8% NaCl) and administered 75 mg/kg/day AST for six weeks, revealing a decrease in mean arterial pressure (MAP) and heart rate in hypertensive rats [132].

However, AST’s ability to alleviate hypertension in clinical trials appears less pronounced. A meta-analysis of 14 randomized controlled trials conducted in 2020 by Xia et al. examined the impact of AST on blood pressure [59]. The results revealed no significant effect of AST on systolic blood pressure (SBP) and diastolic blood pressure (DBP) in healthy subjects, elderly individuals, patients with mild hyperlipidemia, or those with type 2 diabetes mellitus (T2DM). Furthermore, a new meta-analysis performed by Leung et al. in 2022 utilized seven double-blind, randomized controlled trials (RCTs) and indicated that AST had a modest effect on SBP only when the intervention duration exceeded 8 weeks, while no significant influence was observed on DBP [25]. Currently, limited positive evidence exists. Mashhadi et al. showed that 8 mg/day AST supplementation for eight weeks reduced SBP in type 2 diabetic patients [85].

The mechanism underlying AST ameliorated hypertension may involve multiple aspects. Blood flow velocity and the degree of vasodilation can directly impact vascular resistance, and increased vascular resistance may lead to hypertension [133]. In SHRs, AST induced notable histological changes and reduced aortic stiffness to lower SBP [134,135,136]. Hussein et al. demonstrated that ASTs reduced plasma nitric oxide (NO) levels in rats. NO is known to increase cyclic guanosine monophosphate and induce smooth muscle relaxation [134]. AST reduced wall thickness, widened arterial lumens, and decreased aortic elastin, providing novel insights into its potential antihypertensive mechanism in SHRs [137]. In addition, evidence suggests that AST can alleviate hypertension by modulating the renin–angiotensin system. Gao et al. pointed out that the overactivation of the renin–angiotensin system (RAS) leads to hypertension [132]. The classical RAS pathway includes angiotensin II (Ang II), Angiotensin-Converting Enzyme (ACE), and angiotensin II type 1 receptor (AT1R). Experimental results from SHRs demonstrated that AST significantly reduced the expression of ACE and AT1R, indicating that AST can ameliorate hypertension by inhibiting the RAS [138].

**Table 4:** Summary of the effects of AST on hypertension.

Study	Model	Dose of AST	Duration of Intervention	Outcomes	Mechanism
**ANIMALS**					
Gao H., 2021 [138]	Male SHRs and WKY rats	240 mg/day	4 weeks	Blood pressure ↓	Renin–angiotensin system (RAS) in the hypothalamic paraventricular nucleus (PVN) ↑
Gao H., 2021 [132]	Dahl salt-sensitive (S) rats	75 mg/kg/day	6 weeks	Mean arterial pressure ↓ Heart rate ↓	phosphorylated ERK 1/2, phosphorylated JNK ↓
Chen Y., 2020 [135]	SHRs	200 mg/kg/day	11 weeks	DBP ↓ SBP ↓	Proliferating cell nuclear antigen ↓ ki67 ↓
Xuan R., 2016 [139]	Female preeclamptic SD rats	5, 15, 25 mg/kg/day	3 weeks	DBP ↓ SBP ↓	/
Sasaki Y., 2011 [140]	Hypertensive rats	300, 600 mg/kg/day	3 weeks	SBP ↓	NO metabolites ↑ NO_2_/NO_3_ ↑
Hussein G., 2005 [137]	Spontaneously hypertensive rat and stroke-prone SHR-SP strains	50 mg/kg/day	2, 5 weeks	Blood pressure in SHR ↓ Blood pressure in stroke-prone SHR ↓	NO synthesis ↑ Endothelium-dependent and endothelium-independent ↑
**CLINICAL TRIALS**					
Kato T., 2020 [141]	Adult patients with HF due to ischemic or non-ischemic cardiomyopathy	12 mg/day	3 months	Blood pressure ↓ Heart rate ↓	/
Mashhadi N., 2018 [85]	Participants with definitive diagnosis of type 2 diabetes with no insulin therapy	8 mg/day	8 weeks	SBP ↓	/

↑: Up-regulated; ↓: Down-regulated.

## 8. AST and Hyperuricemia

Hyperuricemia (HUA) constitutes a crucial component of MS, arising from abnormalities in human purine metabolism or uric acid (UA) excretion. In MS patients, serum UA levels are markedly elevated, typically exceeding 7 mg/dL [142,143]. As shown in Table 5, AST has demonstrated excellent urate-lowering effects in animal studies. Zhuang et al. administered 25, 50, or 100 mg/kg/day AST to hyperuricemia ICR mice for 14 days and revealed a significant reduction in serum UA content and improvement in renal function markers [144]. Similarly, Le et al. observed that 10, 20, or 40 mg/kg/day AST given orally by gavage to fructose-induced hyperuricemia rats for 6 weeks significantly reduced serum UA levels and enhanced UA excretion via urine [145]. Peng et al. further demonstrated that intra-articular injection of 50 μL of 50 μM AST into the knees of rats successfully inhibited monosodium urate crystal-induced knee joint inflammation [146]. However, clinical trials investigating AST’s effect on HUA are limited. A randomized trial conducted by Klinkenberg et al. involving 32 healthy male cyclists found no significant effect of 4 weeks of 20 mg/day AST intake on serum UA levels [147].

AST prevents the occurrence of HUA in multiple ways. Firstly, AST can inhibit the activity of two key enzymes in the UA synthesis pathway, namely xanthine oxidase (XOD) and adenosine deaminase (ADA), thereby inhibiting UA synthesis at its source [144,145]. Secondly, UA is excreted through urine, a process involving renal reabsorption and secretion, with approximately 90% of UA being reabsorbed back into the bloodstream [148]. The renal excretion of UA is modulated by urate transporters [149,150]. The interplay between UA re-absorption and secretion in the kidney is mediated by various transporters, notably Uric Acid Transporter 1 (URAT1) and GLUT9 [151]. Evidence suggests that AST may reduce serum UA levels by downregulating the mRNA and protein expression of GLUT9 and URAT1 in rats, thereby diminishing UA reabsorption. Le et al. quantified the relative expression of these transporters and found that AST significantly decreased the expression of renal resorptive transporters (GLUT9 and URAT1) in rats [145]. In addition, renal secretory protein Organic Anion Transporter 1 (OAT1) and Organic Anion Transporter 3 (OAT3) are primarily involved in secreting UA into the proximal renal tubule, specifically via the basolateral membrane into epithelial cells [152,153]. Le et al. pointed out that AST significantly upregulated the expression of OAT1 and OAT3 in the kidneys of hyperuricemia rats to facilitate UA clearance [145]. ABCG2, a high-capacity urate secretory transporter, exhibits a close correlation between its activity and serum UA levels [154]. Yano et al. examined various ABCG2 transporter mutants and found that decreased urate transporter activity was proportional to reduced ABCG2 protein expression [155]. Dysfunctional ABCG2 significantly elevates the risk of HUA and even gout, particularly in younger individuals [149]. Le et al. pointed out that AST significantly upregulated the expression of ABCG2 in the kidney of hyperuricemia rats to facilitate UA clearance [145].

More studies are needed to elucidate the urate-lowering effects of AST in human trials and explain the exact mechanisms by which AST reduces uric acid.

**Table 5:** Summary of the effects of AST on hyperuricemia.

Study	Model	Dose of AST	Duration of Intervention	Outcomes	Mechanism
**ANIMALS**					
Zhuang J., 2021 [144]	Male ICR mice	25, 50, 100 mg/kg/day	14 days	Serum UA ↓	XOD and ADA ↓ NF-κB/NLRP3 pathways ↓
Le Y., 2020 [145]	Rats	10, 20, 40 mg/kg/day	40 days	Serum UA ↓ Urine UA ↑ 24 h Total UA Excretion ↑	XOD and ADA ↓ GLUT9 and URAT1 ↓ OAT1, OAT3 and ABCG2 ↑
**CLINICAL TRIALS**					
Klinkenberg L., 2013 [147]	32 well-trained male cyclists	20 mg/day	4 weeks	Serum UA -	/

↑: Up-regulated; ↓: Down-regulated; -: Have no significant impact.

## 9. Conclusions

MS is primarily manifested by symptoms such as obesity, hyperglycemia, dyslipidemia, hypertension, and hyperuricemia. AST, an excellent functional substance derived from natural sources, exhibits improvement in various components of MS. This article reviews the progress of research on the ameliorative effects and mechanisms of AST on the primary symptoms of MS, as well as some current shortcomings in the research. In general, AST exhibits attractive efficiency in animal models to improve obesity, hyperglycemia, dyslipidemia, hypertension, and hyperuricemia. Clinical trials basically verified the ameliorative effects of AST on hyperglycemia and dyslipidemia, while the results on obesity and hypertension are moderate. Limited human studies were conducted on the efficiency of AST on hypertension. The underlying mechanisms were partially revealed by various evidence. For instance, AST activates the AMPK pathway and serves as a typical PPARα agonist, enabling it to regulate energy metabolism and promote lipid metabolism in the body, thereby alleviating obesity, hyperglycemia, and dyslipidemia related to MS. Additionally, AST improves insulin sensitivity and accelerates glucose metabolism through the activating of PI3K/AKT pathway. The beneficial effects of AST on intestinal flora and their metabolites can also explain the ameliorative effects on hyperglycemia and dyslipidemia. By inhibiting angiotensin systems and modulating plasma NO levels, AST alleviates hypertension. AST reduces blood uric acid by inhibiting uric acid synthesis and decreasing uric acid re-absorption. However, current research on AST still faces limitations. Importantly, the potential differences in effects and mechanisms among various AST isomers are often overlooked, with a lack of discrimination of AST structures and a default assumption of the all-*E* form. Furthermore, research on AST derivatives, such as AST esters and astacene, remains inadequate. Additionally, more well-designed clinical trials with appropriate dosages and duration are necessary to elucidate the roles of AST in alleviating MS-related components in humans. Moreover, the poor water solubility, stability, and low bioavailability of AST are noteworthy. In the future, more studies on encapsulation and targeted delivery systems are needed to address the defects of AST and improve its application in food and pharmaceuticals.

## Data Availability

Not applicable.
